# A new species of *Gordius* (Phylum Nematomorpha) from terrestrial habitats in North America

**DOI:** 10.3897/zookeys.892.38868

**Published:** 2019-11-27

**Authors:** Christina Anaya, Andreas Schmidt-Rhaesa, Ben Hanelt, Matthew G. Bolek

**Affiliations:** 1 Department of Integrative Biology, 501 Life Sciences West, Oklahoma State University, Stillwater, Oklahoma 74078, USA Oklahoma State University Stillwater United States of America; 2 Zoological Museum and Institute, Biocenter Grindel, Martin-Luther-King-Platz 3, University of Hamburg, 20146 Hamburg, Germany University of Hamburg Hamburg Germany; 3 Center for Evolutionary and Theoretical Immunology, Department of Biology, 163 Castetter Hall, University of New Mexico, Albuquerque, New Mexico 87131-0001, USA University of New Mexico Albuquerque United States of America

**Keywords:** eggs, Gordiida, hairworm, life cycles, North America, Oklahoma, soil

## Abstract

Freshwater hairworms (class Gordiida) are members of the phylum Nematomorpha that use terrestrial arthropods as definitive hosts but reside as free-living adult worms in rivers, lakes, or streams. The genus *Gordius* consists of 90 described species, of which three species were described from freshwater habitats in North America. In this paper we describe a new species of *Gordius* from terrestrial habitats in Oklahoma, Texas, and Louisiana, United States. Oddly, each year hundreds of adult free-living worms appear after bouts of heavy rain on streets, sidewalks, and lawns during the winter season, when terrestrial arthropod hosts are not active. The new species is described based on morphological characters of adults and non-adult stages including the egg strings, eggs, larvae, and cysts. Adult males have a unique row of bristles on the ventral inner side of each tail lobe and a circular pattern of bristles on the terminal end of each lobe, which distinguishes them from all other described North American species of *Gordius*. The egg string, larval, and cyst morphology of this new species conform to previous descriptions of non-adult hairworm stages for the genus *Gordius*. However, the eggs of this new species of hairworm are unique, as they contain an outer shell separated by distinct space from a thick inner membrane. The consistent occurrence of this gordiid in terrestrial habitats, along with its distinct egg morphology, suggests that this new species of hairworm has a terrestrial life cycle.

## Introduction

The Phylum Nematomorpha, commonly known as hairworms or Gordian worms, or simply gordiids, are parasites of terrestrial arthropods with a complex life cycle that includes a free-living and parasitic phase with multiple hosts (Carvalho 1942; Townsend 1970; Blair 1983; Poinar and Brockerhoff 2001; Hanelt et al. 2005). However, their short lifespan, cryptic coloration, and hiding behavior makes hairworms difficult to collect for biodiversity studies (May 1919; Hanelt et al. 2015). An analysis of all known life cycles indicates that juvenile gordiids infect terrestrial arthropods from which free-living adults emerge into freshwater habitats, such as streams, rivers, and lakes (May 1919; Hanelt et al. 2005; Bolek et al. 2015). After emerging from their arthropod host, dioecious species mate and females deposit egg strings in aquatic habitats (Bolek et al. 2013). Within weeks, larvae develop, hatch, infect, and encyst indiscriminately within a variety of aquatic vertebrate and invertebrate animals (Hanelt and Janovy 2003). Some of these infected animals, such as aquatic insect larvae, act as paratenic (transport) hosts by carrying cysts to land where they are consumed by omnivorous or predatory definitive hosts including millipedes, crickets, beetles, cockroaches, and mantids (Bolek et al. 2015).

Although first described more than 300 years ago, gordiids have been identified as one of the most understudied groups of parasites (Poulin 1998). Currently, it is hypothesized that only 18% of the estimated 2000 gordiid species have been described (Bolek et al. 2015). Because of their life cycle that includes an aquatic environment where worms emerge as free-living adults from their arthropod host, sampling for hairworms and discovering their true biodiversity has been challenging (Hanelt et al. 2005). However, during the last 15 years, advances in sampling, culturing, and barcoding techniques for gordiids have resulted in the descriptions of more than 50 new species including a parthenogenetic species (Bolek et al. 2010; Schmidt-Rhaesa and Prous 2010; Hanelt et al. 2012; Bolek et al. 2013a; Chiu et al. 2017; Swanteson-Franz et al. 2018).

At present, approximately 360 gordiid species have been described from across the world within 18 extant and two extinct genera (Schmidt-Rhaesa 2013; Bolek et al. 2015; Yadav et al. 2018). Of those, the genus *Gordius* Linnaeus, 1758 is the second largest in terms of described species, with 90 valid species distributed across the world (Schmidt-Rhaesa 2010, 2013). The diagnostic characters for the genus *Gordius* are based on male characteristics and include a semicircular or parabolic cuticular fold posterior of the cloacal opening, known as the postcloacal crescent, and a bilobed posterior end with rounded posterior tips. The posterior end of females is rounded, with a terminal cloacal opening. The anterior end is distinctly tapering, with a white tip, known as a calotte, followed by a brown or black collar usually present in both sexes of most species. Additionally, various combinations of a dark ventral and/or dorsal line, and/or white spots on the cuticle are often present on free-living male and/or female worms of several species (Schmidt-Rhaesa 2010). However, compared to other gordiid genera, the genus *Gordius* contains few cuticular structures, such as areoles, that demonstrate intraspecific and interspecific variability making species identification difficult (Schmidt-Rhaesa 2013).

The majority of *Gordius* diversity has been identified from the palearctic region which harbors 71% of *Gordius* diversity with the remaining 29% distributed throughout the world with the exception of Antarctica (Schmidt-Rhaesa 2010). Currently three valid species of *Gordius* have been described from the Nearctic region representing 4% of *Gordius* species. These include *Gordius attoni* Redlich, 1980, *Gordius difficilis* Smith, 1994, and *Gordius robustus* Leidy, 1851 (Schmidt-Rhaesa et al. 2003; Schmidt-Rhaesa 2010, 2013; Schmidt-Rhaesa et al. 2016). Of those, *G. robustus* is one of the most commonly reported and widely distributed hairworm species in North America (Schmidt-Rhaesa et al. 2003; Schmidt-Rhaesa 2010). However, recent sampling efforts across North America for *G. robustus*, combined with molecular data indicate that *G. robustus* is a complex of at least eight distinct species (Hanelt et al. 2015).

Based on genetic data, one of the eight species, identified as clade 7 by Hanelt et al. (2015), occurs in Oklahoma, Texas, and Louisiana. In this article, we describe free-living adults of this new species of *Gordius* collected from locations in Oklahoma Texas, and Louisiana using light and scanning electron microscopy. In addition, we describe the non-adult life stages, including the egg strings, eggs, larvae and cysts. Finally, based on morphological characteristics of non-adult stages, and the occurrence of adult free-living worms of this new species in terrestrial habitats, we provide evidence and suggest that this new species of gordiid has a terrestrial life cycle.

## Materials and methods

### Field collections

A total of 39 female and 194 male free-living hairworms were collected from two suburban locations in the city of Stillwater, OK, USA (36.12091, -97.03669; 36.13653, -97.04266). All free-living worms were collected after bouts of heavy rain from streets, sidewalks, or lawns between November-December 2014 and January-March 2015. In addition, each location was searched for potential definitive arthropod hosts by visually scanning the locations when worms were present. All specimens were placed in 950 ml glass jars containing aged tap water and transported to the laboratory at Oklahoma State University. A subsample of adult worms was processed for morphological characters; whereas the remaining worms were allowed to mate to obtain non-adult life stages (see below). Additionally, two male specimens from a single location in Montgomery, Texas (30.38988, -95.69552) and one male from Baton Rouge, Louisiana (30.40661, -91.18734) were collected by citizen scientists and sent to us as per the instructions on our website (www.nematomorpha.net) and its Report-A-Worm feature.

### Biological material and microscopy

**Adults.** Length, width, color, and color pattern (presence of a calotte, dark pigmented ring, and spots on the cuticle) were recorded for all male and female individuals collected from Stillwater, OK. Lengths of worms were obtained by placing individuals on a ruler without stretching the specimen and measured to the nearest 1 mm. The width of each worm was obtained using an Olympus SZ1145 Stereomicroscope and a calibrated ocular micrometer. Posterior ends of males were then photographed with a Sony Cybershot camera and the angle of postcloacal crescent was measured using ImageJ software (Schneider et al. 2012).

For scanning electron microscopy (SEM), four female and six male worms collected from Oklahoma and two males collected from Texas were imaged as described by Harkins et al. (2016). Briefly, live worms were preserved in 70% ethanol at room temperature and 5–10 mm sections of the anterior, posterior, and mid-body regions of each worm were cut with a razorblade. Specimens were then dehydrated in increasing concentrations of ethanol (70 %, 85 %, 95 %, 100 %), dried using hexamethyldisilazane (HDMS) according to Harkins et al. 2016, mounted on aluminum stubs, sputter coated with gold palladium, and examined with an FEI Quanta 600 field emission gun ESEM (ThermoFisher Scientific, Hillsboro, OR) with Evex EDS and HKL EBSD or a JEOL 5800LV SEM at 15 kV (JEOL Ltd., Tokyo, Japan). All terminology for adult worms follows Schmidt-Rhaesa (2010).

**Obtaining non-adult stages.** A subset of single male and female worms from Stillwater, OK were paired and placed in 110 × 35 mm Stender dishes filled with filtered and aged tap-water (Szmygiel et al. 2014). Observations were made daily on the mating and oviposition behavior of worms. After males deposited a sperm drop on the posterior end of females, females were isolated and allowed to deposit eggs strings in individual Stender dishes filled with aged tap-water. Egg strings were rinsed in a solution of 1-part 5.25 % chlorine bleach to 250-parts water to prevent fungal growth and visually observed over a period of 2–5 weeks for larval maturation, indicated by a color change in egg strings from white to yellow in color. After hatching a subset of larvae was pipetted into 0.2 mL microtubes and stored at -80 °C for snail infections according to Bolek et al. (2013b). To obtain cysts, *Physa acuta* (Draparnaud, 1805) snails were reared in the laboratory according to Szmygiel et al. (2014). A subset of hatched larvae was thawed, collected with a Pasteur pipette and approximately 100–200 larvae were pipetted into 48, 1.5 ml well-plates filled with 1 mm of aged tap water. A single laboratory reared *Physa acuta* snail was then added to each well. Snails fed on the larvae mixture for 48 hours and snails were then maintained in 3.75 L jars filled with aerated aged tap water and fed on a diet of frozen lettuce and Tetra Min fish food for a period of four weeks. To evaluate cyst development, every week for a period of four weeks post infection (WPI), a subsample of snails was placed in labeled and capped 50 ml plastic centrifuged tubes, filled with approximately 35 ml of aged tap water, and frozen at -80 °C following the protocol of Bolek et al. (2013b). The gastropod nomenclature is according to Wethington and Lydeard (2007).

**Morphology of egg strings, eggs, and larvae.** Photographs were taken of two-day old egg strings in Stender dishes and a plastic ruler as a reference using a Sony Cybershot camera and the length and width of 20 egg strings was measured using ImageJ software. Individual developed eggs, and two-day old larvae after hatching were prepared as live wet mounts and observed using an Olympus BX–51 upright research microscope (Olympus, Tokyo, Japan) configured for bright field and Nomarski differential interference contrast (DIC) microscopy with plain fluorite objectives at 400× to 1000× total magnification. Measurements of developed eggs with larvae were taken from captured digital images using an Olympus 5–megapixel digital camera and ImageJ software. Briefly, for developed eggs, 5 mm sections of egg strings were placed on microscope slides in a drop of water, covered with a coverslip without crushing, and observed for general morphology with an Olympus BX–51 upright research microscope and the length and width was recorded for 30 eggs. For larvae, the length and width of the preseptum, postseptum, pseudointestine, and stylets was measured for 30 individuals following the protocols of Szmygiel et al. (2014).

**Morphology of cysts.** Laboratory infected and post frozen snails were processed for gordiid cysts following Harkins et al. (2016). Briefly, all frozen snails were thawed, each snail’s body was removed from its shell using a dissection microscope with forceps and pressed between two slides (Harkins et al. 2016). A wet mount was prepared by removing the top slide and adding a drop of water and covering the flattened tissue with a coverslip. Slides were then examined with an Olympus BX–51 microscope as described for eggs and larvae. Thirty cysts were digitally photographed at 1000× total magnification and the length and width of the cyst, cyst wall and encysted larvae were obtained using ImageJ software. Finally, the folding pattern of all encysted larvae was recorded. Procedures and terminology for cyst stages of gordiids follows Hanelt and Janovy (2002), Szmygiel et al. (2014) and Harkins et al. (2016).

**Larval preparation for SEM and larval characters.** Pieces of egg strings with developed larvae and hatched larvae suspended in water, were pipetted onto Poly-L-Lysine coated coverslips placed in 1.5 ml plastic well plates and fixed in a solution of alcohol, formalin, and acetic acid. Fixed larvae were dehydrated in a graded series of ethanol in each plastic well with 0.5 ml of 30 %, 50 % and 70 % ethanol for 30 min each, followed by dripping 1 ml of 100% ethanol into the well over a period of an hour, 1 ml of ethanol was then removed from the well and the process repeated three additional times (Harkins et al. 2016). Finally, specimens were dried using HDMS, mounted on aluminum stubs, coated with gold palladium, and examined with an FEI Quanta 600 field emission gun ESEM with Evex EDS and HKL EBSD housed at Oklahoma State University. The following morphological surface characteristics were recorded for at least 30 individual larvae: number of terminal spines on the postseptum, the number and relative size of cuticular hooks on the preseptum, the proboscis orientation (dorso-ventrally or laterally compressed) and the number and orientation of spines on the proboscis. External morphological characteristics for larvae examined with SEM followed terminology by Szmygiel et al. (2014). All measurements are reported as a mean ± 1 standard deviation followed by the range.

**Egg morphology of aquatic gordiids.** To compare the egg morphology of the new species to eggs of aquatic gordiids, we examined egg photomicrographs from our personal collections for three species/genera of aquatic hairworms. All species examined were collected from streams as free-living adults or cysts that were reared in crickets in the laboratory. These included *Gordius difficilis* from Waukesha County, Wisconsin, USA (42.966229, -88.364328), *Neochordodes occidentalis* Montgomery, 1898 from Pima County, Arizona, USA (31.8655, -109.1905) and *Paragordius obamai* Hanelt, Bolek, and Schmidt-Rhaesa 2012 from Nyanza province, Kenya (-0.1519, 34.4455). Information on how eggs were obtained and processed is reported in Bolek and Coggins (2002), Hanelt et al. (2012) and Szmygiel et al. (2014). Briefly, pieces of egg strings of each hairworm species were placed on a microscope slide with a drop of water and covered with a cover slip. Most eggs were examined using DIC microscopy. However, some eggs were examined with bright field microscopy and in this case, a drop of Nile Blue was added to the wet mount to visualize the inner egg content.

## Taxonomy

### Gordius terrestris sp. nov.

Taxon classificationAnimaliaGordeaGordiidae

FAE62113-6FA0-589A-A5A2-9AFDB4790D54

http://zoobank.org/6A529B7C-147D-450D-B3AA-D3A4E82305AC

#### Type locality.

A suburban lawn in the City of Stillwater, Payne County, Oklahoma; USA (36.12091, -97.03669; approximate altitude: 276–296 m).

***Holotype*.** Male collected on 5 December 2014. Deposited in the Museum of Southwestern Biology (MSB) Parasite Division, University of New Mexico (UNM), New Mexico, USA with accession number MSB:Para:29147.

***Paratypes*.** Female specimen collected on 5 December 2014, from the type locality. Deposited into the MSB Parasite Division, accession number MSB:Para:29148. Paratypes: two males collected 14 January 2003 in Montgomery, Texas (30.38988, -95.69552). Deposited into the MSB Parasite Division, accession numbers MSB:Para:19257 and MSB:Para:19258.

#### Other material deposited.

Larvae and egg strings with hatching larvae on SEM stubs obtained from laboratory cultures from Oklahoma collected worms. Deposited into the Museum of Southwestern Biology (MSB) Parasite Division, http://grbio.org/institution/university-new-mexico (UNM), New Mexico, USA with accession number MSB:Para:29149.

#### Host.

Natural definitive host is unknown, and no arthropod hosts were found during times when adult free-living worms were present.

#### Etymology.

The new species is named after the terrestrial habitat from which all adult free-living individuals were collected.

#### Distribution.

Stillwater, Oklahoma (36.12091, -97.03669; 36.13653, -97.04266), Montgomery, Texas (30.38988, -95.69552) and Baton Rouge, Louisiana (30.40661, -91.18734).

#### Link to molecular data.

GenBank accession numbers for mitochondrial (CO1 and cytb) and ribosomal (partial 28S, ITS1, 5.8S and ITS2) DNA sequences for the Louisiana (KM382307; KM382349; KM382400), Oklahoma (KM382308 and KM382309; KM382350) and Texas (KM382351; KM382401 and KM382402; KM382403 and KM382310; KM382352; KM382404) samples of *G. terrestris* sp. nov. were published in Hanelt et al. (2015) but are provided in this description for consistency.

#### Material examined.

Adults (*N* = 233), eggs, larvae, and cysts. Eight adult males, six from Oklahoma and two from Texas, and four adult females from Oklahoma were imaged using SEM; and other male and female individuals were examined using DIC and bright field microscopy for color pattern. Additionally, egg, larvae, and cyst stages were imaged using SEM and/or DIC microscopy.

#### Description of male.

**Figure 1.**
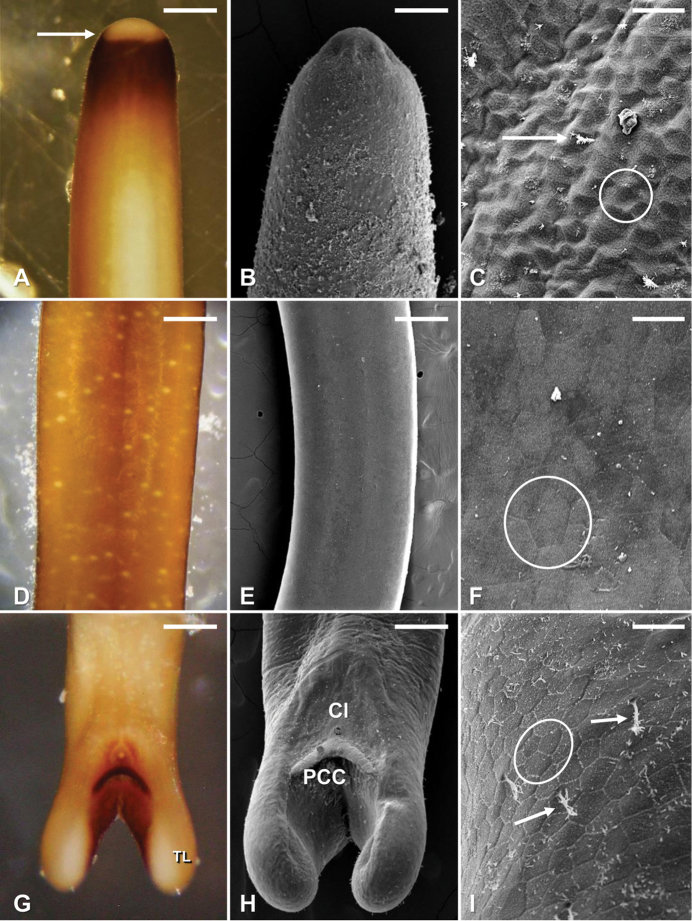
*Gordius terrestris* sp. nov., adult male from Stillwater, Oklahoma, light (**A, D, G**) and SEM (**B, C, E, F, H, I**) photomicrographs **A** anterior body region showing typical color pattern, showing the distinct calotte (arrow) and dark ring **B** anterior end, dorsal view **C** areole pattern on the anterior body region. Note the weakly developed areoles (circle) and the presence of bristles (arrows) **D** midbody region, dorsal view, showing distinct white spots and medial line **E** Midbody region, dorsal view, showing typical cuticular pattern **F** areole pattern on the midbody region; note the weakly developed polygonal shaped areoles (circle) **G** posterior body region, ventral view, showing distinct coloration; note the darkly pigmented postcloacal crescent and dark pigmentation on inner sides of the tale lobes (TL) **H** ventral view of the posterior region, showing the cloaca (Cl) and postcloacal crescent (PCC) **I** areole pattern on the posterior body region; note the weakly developed polygonal shaped areoles (circle) and the bristles (arrows). Scale bars: 210 µm (**A**); 130 µm (**B**); 18 µm (**C**); 220 µm (**D, G**); 290 µm (**E**); 10 µm (**F**); 175 µm (**H**); 20 µm (**I**).

**Figure 2.**
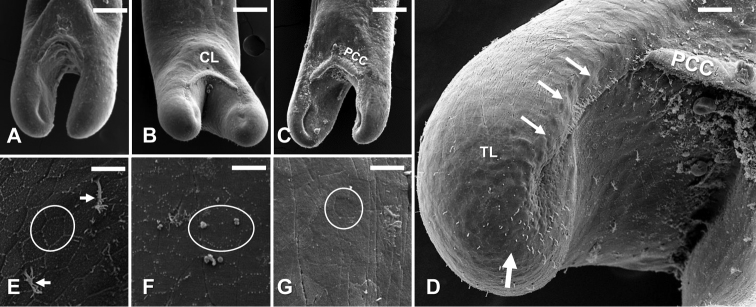
*Gordius terrestris* sp. nov., adult males from Stillwater, Oklahoma, SEM photomicrographs **A–C** posterior body region, ventral view, note the variation in the shape of tail lobes and postcloacal crescents (PCC) below the cloaca (CL) **D** tail lobe showing the distinct row of bristles beginning below the postcloacal crescent (PCC) and progressing on the ventral inner side (small arrows) of the tail lobe (TL); and bristles distributed in a circular pattern on the terminal end (large arrow) of the tail lobe **E–G** variation in the weakly developed polygonal shaped areoles (circles) on the posterior body region of different male individuals; note the branching bristles (arrows) in **E**. Scale bars: 175 µm (**A–C**); 75 µm (**D**); 8 µm (**E–G**).

Adult free-living males were creamy white to dark brown in color and contained distinct white spots throughout the length of the body (Fig. 1D). A dark dorsal and ventral medial line was present along the length of the cuticle being most distinct in the mid-body region (Fig. 1D). Males were 258 ± 73 (122–470; *N* = 194) mm in length and 0.6 ± 0.1 (0.4–0.9) mm in width. The anterior end was tapered and contained a white calotte followed by a dark collar (Fig. 1A, B). The cuticle was variable among individuals but contained one type of areole distributed on the anterior, midbody, and/or posterior regions of the body with various bristles distributed among the areoles (Figs 1C, F, I; 2E–G). Areoles were weakly developed, polygonal in shape, and 9–12 μm in diameter (Figs 1C, F, I; 2E–G). The posterior end of males contained two terminal tail lobes which were 0.50 ± 0.1 (0.4–0.7) mm long and 0.2 ± 0.04 (0.17–0.3 mm) wide (Figs 1G, H; 2A–D). Each tail lobe contained a distinct row of bristles on the ventral inner side and distinct bristles distributed in a circular pattern on the terminal ends of each lobe (Fig. 2D). Additionally, the inner side of the lobes were darkly pigmented compared to the lighter creamy white color of each lobe (Fig. 1G). The cloacal opening was round and situated ventrally in a broad nonareolar field above the postcloacal crescent (Figs 1G, H; 2B, C). The postcloacal crescent was situated between the proximal ends of the two tail lobes and was dark brown in color (Fig. 1G) and had an angle of 111 ± 9° (102–126°) (see Figs 1G, H; 2B–D).

#### Description of female.

**Figure 3.**
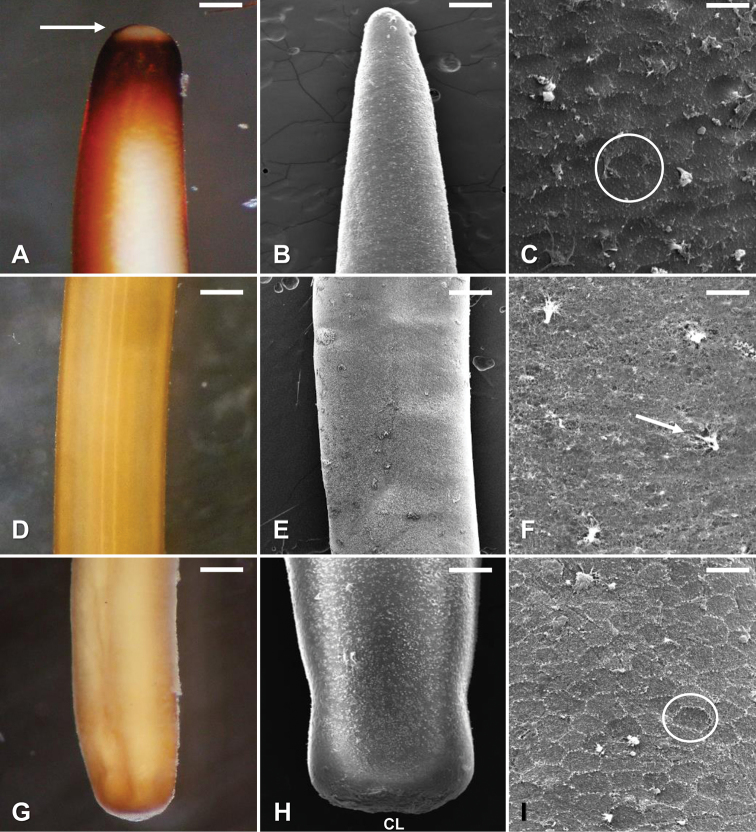
*Gordius terrestris* sp. nov., adult females from Stillwater, Oklahoma, light (**A, D, G**) and SEM (**B, C, E, F, H, I**) photomicrographs **A** anterior body region showing typical color pattern, showing the distinct calotte (arrow) followed by a dark ring **B** anterior end, dorsal view **C** areole pattern on the anterior body region; note the weakly developed polygonal shaped areoles (circle) **D** midbody region, lateral view, showing typical color pattern **E** midbody region, dorsal view showing typical cuticular pattern **F** midbody region, dorsal view, showing finer details of the cuticle; note the branching bristles (arrow) **G** posterior body region, ventral view, showing typical coloration **H** posterior body region, ventral view showing the location of the cloaca (CL) **I** posterior body region, areole pattern on the posterior body region; note the weakly developed polygonal shaped areoles (circle). Scale bars: 160 µm (**A**); 150 µm (**B**); 10 µm (**C**); 440 µm (**D, G**); 330 µm (**E**); 15 µm (**F, I**); 190 µm (**H**).

Adult free-living females were creamy white to dark brown, and contained dark dorsal and ventral lines along the length of the body. Females were 246 ± 41 (211–336; *N* = 39) mm long by 1.0 ± 0.1 (0.7–1.3) mm wide. The anterior end was tapered and contained a white calotte followed by a dark collar (Fig. 3A, B). Areoles were weakly developed, polygonal in shape, and 11–13 μm in diameter with branching bristles being scattered across the cuticle (Fig. 3C, F, I). The posterior end of females was round and cylindrical in shape and darkly pigmented on the terminal end (Fig. 3G, H). The cloaca was round in shape and located on the terminal end.

#### Description of mating, oviposition, egg strings, and eggs.

**Figure 4.**
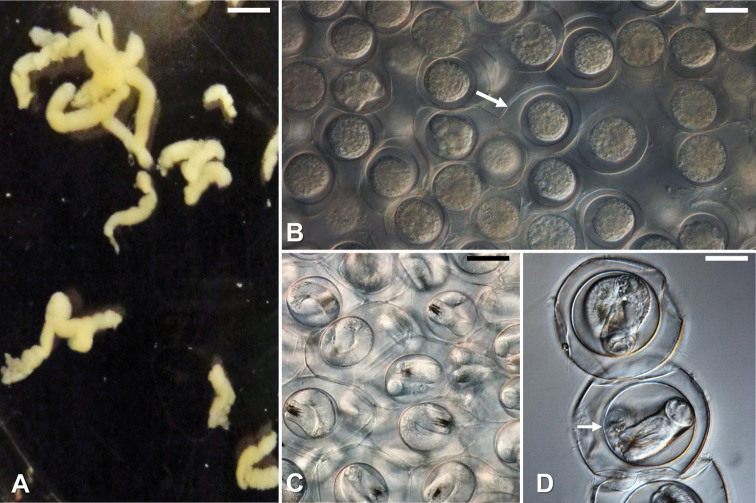
*Gordius terrestris* sp. nov., eggs and egg strings, light photomicrographs **A** newly deposited egg strings **B** egg string segment showing tightly aggregated undeveloped eggs; note the eggshell (arrow) **C** segment of an egg string showing developing larvae within eggs **D** eggs with fully developed larvae; note the distinct space between the eggshell and the thick inner membrane. Scale bars: 4 mm (**A**); 40 µm (**B**); 25 µm (**C**); 20 µm (**D**).

When placed together, male and female worms immediately formed Gordian knots. Males moved up and down the female’s body with their coiled posterior end. Once the male’s bifurcated tail was in proximity of the female’s cloaca, the male deposited a mass of sperm on the female’s posterior end. Egg strings were deposited within 7–30 days after copulation. Newly deposited egg strings were white in color and deposited in a continuous string that broke as it emerged from the female’s cloaca into short segments (Fig. 4A). Deposited egg strings were 7 ± 4 (2–19) mm in length and 1.2 ± 0.3 (0.8–1.9) mm in width. Over two to three weeks the white eggs strings darkened to a tan color and contained fully developed larvae within eggs (Fig. 4C, D). Developed eggs were tightly aggregated together within egg strings and spherical to elliptical in shape (Fig. 4B, C). Eggs were 55 ± 7 (42–72) µm long by 55 ± 7 (43–68) µm wide. Each egg contained an outer shell separated by distinct space from a thick inner membrane (Fig. 4B–D). The distinct inner membrane was 38 ± 3 (29–42) µm long by 39 ± 4 (30–45) µm wide.

#### Description of larvae.

**Figure 5.**
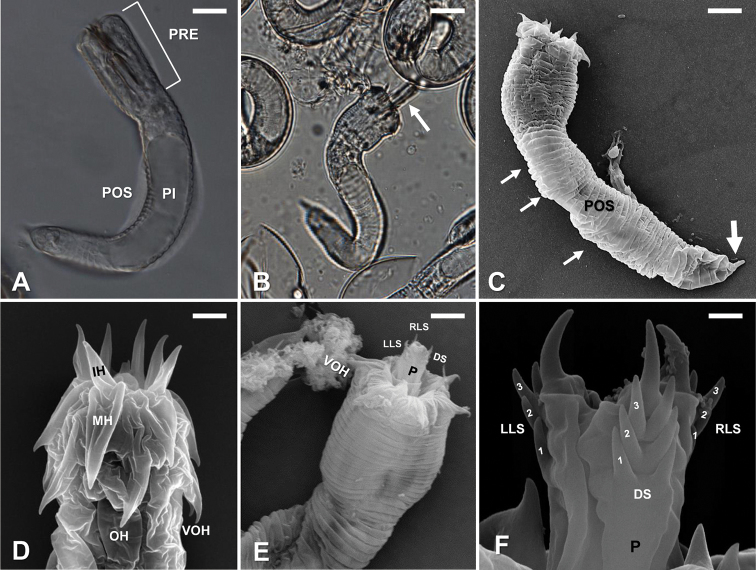
*Gordius terrestris* sp. nov., larvae, light (**A, B**) and SEM (**C–F**) photomicrographs **A** live larva, showing the preseptum (PRE), postseptum (POS) and pseudointestine (PI) **B** recently hatched larvae showing everted proboscis (arrow) **C** larva note the superficial annulations (small arrows) and a single terminal spine located (large arrow) on the posterior region of the postseptum (POS) **D** preseptum, showing the arrangement of three sets of cuticular hooks, including outer hooks (OH), middle hooks (MH) and inner hooks (IH); and fused ventral outer hooks (VOH) **E** anterior end with the eversible proboscis (P); note the distinct spines on the distal end of the left lateral side (LLS), right lateral side (RLS) and dorsal side (DS) in respect to the ventral outer hooks (VOH) **F** partially everted proboscis (P) showing pairs of small spines (numbers) and a larger terminal spine on the distal end of the left lateral (LLS), right lateral (RLS) and dorsal sides (DS). Scale bars: 12 µm (**A**); 13 µm (**B**); 8 µm (**C**); 2.5 µm (**D**); 6 µm (**E**) 0.8 µm (**F**).

Larvae of *G. terrestris* sp. nov. possessed a cylindrical body divided by a septum into two regions, the preseptum and a postseptum (Fig. 5A, B). The preseptum was 30 ± 6 (22–40) µm in length and 20 ± 2 (16–26) µm in width and contained an eversible proboscis supported with three internal stylets which were 17 ± 4 (10–25) µm in length and 5 ± 1.3 (2–8) µm in width (Fig. 5B). The postseptum was 106 ± 12 (76–127) µm in length and 20 ± 18 (15–23) µm in width and contained a clearly visible pseudointestine. The pseudointestine was an elongated oval structure, subdivided into two portions (Fig. 5A). The pseudointestine was 80 ± 10 (57–104) µm in length and 12 ± 2 (10–17) µm in width.

Externally, larvae were superficially annulated with a single spine located on the posterior region of the postseptum (Fig. 5C). The preseptum had three sets of cuticular hooks (Fig. 5D). The outer ring of hooks contained seven hooks, two of which were fused proximally and located on the ventral side (Fig. 5D). The middle and inner rings contained six hooks each (Fig. 5D). The eversible proboscis contained three pairs of spines and one terminal spine on the distal end of the left lateral, right lateral and dorsal sides (Fig. 5E, F).

#### Cyst development and morphology.

**Figure 6.**
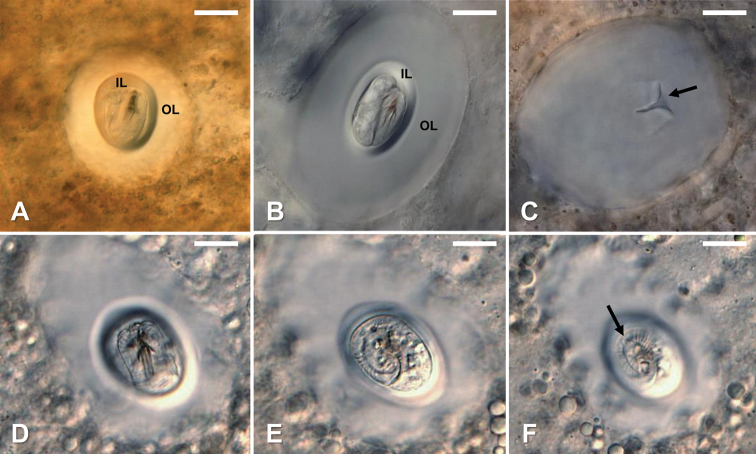
*Gordius terrestris* sp. nov., cysts, light photomicrographs **A–B** fully formed cysts in experimentally infected *Physa acuta* snails; note the folded larva surrounded by a clear cyst wall of unknown composition with a distinct inner layer (IL) and outer layer (OL) **C** remaining cyst wall after the folded larvae was extruded under coverslip pressure. Note the opening where the larvae emerged (arrow) **D–F** different focal planes showing the distinct larvae folding pattern; note the location of the terminal spine (arrow) in F and that the larva folds twice within the fully formed cyst. Scale bars: 20 µm (**A–F**).

After being ingested by snails, larvae develop into cysts and became distributed throughout the snail tissues. During cyst formation the content of the larval pseudointestine was emptied and larvae folded their postseptum twice around the preseptum (Fig. 6D–F). The posterior end of the postseptum always reached the posterior end of the preseptum and protruding spines were never visible on the anterior end of fully formed cysts (Fig. 6A, B). Fully formed cysts of *G. terrestris* sp. nov. were observed in laboratory exposed snails 2–3 WPI and possessed a clear cyst wall of unknown composition with a distinct inner layer surrounding the folded larva (Fig. 6A, B). Cysts were 102 ±16.7 (68–131) µm in total length and 101 ± 13 (72–140) µm in total width (Fig. 6B). Folded larvae inside of the cyst were 29 ± 7 (17–39) µm in length and 31 ± 5 (18–43) µm in width.

#### Diagnosis and taxonomic comments.

*Gordius terrestris* sp. nov. has unique morphological features which warrant placing it as a new species and make it distinct from the other three described Nearctic species of *Gordius*. *Gordius terrestris* sp. nov. differs morphologically from *G. difficilis* by lacking distinct pre-cloacal bristles which are present in males of *G. difficilis* (Bolek and Coggins 2002). Additionally, *G. terrestris* sp. nov. has distinct polygonal areoles and therefore differs morphologically from the description of *G. robustus* which has a smooth cuticle (Schmidt-Rhaesa et al. 2003). Although, *G. attoni* and *G. terrestris* sp. nov. both have polygonal shaped areoles and distinct white spots on the cuticle of males, *G. attoni* areoles contain microscopic processes which are absent on the areoles of *G. terrestris* sp. nov. (Redlich 1980; Schmidt-Rhaesa et al. 2003). In addition, male *G. terrestris* sp. nov. contain an aggregation of bristles on the ventral inner side of each tale lobe posterior of the postcloacal crescent and distinct bristles distributed in a circular pattern on the terminal ends of each lobe, which are not present in male *G. attoni*, *G. difficilis* or *G. robustus* (Redlich 1980; Smith 1994; Bolek and Coggins 2002; Schmidt-Rhaesa et al. 2003; Schmidt-Rhaesa 2010). Finally, published molecular data by our group on *G. terrestris* sp. nov., *G. attoni* and seven other undescribed species of *Gordius* collected across the United States and one undescribed species from Mexico, indicate that *G. terrestris* sp. nov. (described as clade 7) is genetically distinct from all other *Gordius* species for which genetic data are available (Hanelt et al. 2015). Mitochondrial CO1 genetic distances indicate that *G. terrestris* sp. nov. differs by 8–21 % in the CO1 genetic distance from the other seven undescribed species of *Gordius* from the United States and one from Mexico and by 17% from *G. attoni*, but only differs by 1.5 % within individuals collected from Oklahoma, Texas, and Louisiana (Hanelt et al. 2015).

Although, the distribution of the cuticular bristles on tail lobes of male *G. terrestris* sp. nov. distinguish it from all other described North American species of *Gordius*, several European species including *Gordius helveticus* Schmidt-Rhaesa, 2010, *Gordius karwendeli* Schmidt-Rhaesa, 2010, *Gordius spiridonovi* Spiridonovi, 1984, *Gordius terminosetosus* Schmidt-Rhaesa, 2010, and *Gordius zwicki* Schmidt-Rhaesa, 2010 also contain cuticular bristles on tail lobes (Schmidt-Rhaesa 2010; Schmidt-Rhaesa and Prous 2010). Of those, the distribution pattern of cuticular bristles on tail lobes of male *G. terrestris* sp. nov. is most similar to *G. helveticus*. However, *G. helveticus* lacks well-defined areoles and therefore can be easily distinguished from *G. terrestris* sp. nov. (Schmidt-Rhaesa 2010).

**Figure 7.**
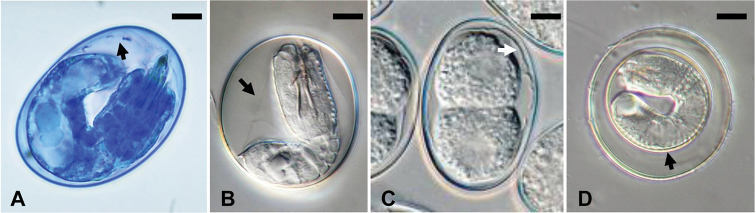
Eggs of aquatic and terrestrial hairworms, light photomicrographs **A** egg of *Gordius difficilis* stained with Nile Blue; note the thin inner membrane (arrow) surrounding the larva **B** egg of *Neochordodes occidentalis* showing a thin inner membrane (arrow) surrounding the larva **C** egg of *Paragordius obamai* showing a developing larva surrounded by a thin inner membrane (arrow) **D** egg of *Gordius terrestris* sp. nov. showing a distinct thick inner membrane (arrow) surrounding the larva. Scale bars: 6 µm (**A**); 8 µm (**B, C**); 11 µm (**D**).

The general morphology of the egg string, larvae, and larval folding pattern within the cysts of *G. terrestris* sp. nov. conform to previous descriptions of these non-adult stages for the genus *Gordius*. However, these non-adult stages are morphologically distinct from egg strings, larvae, and cysts of other gordiid genera such as *Chordodes*, *Neochordodes* and *Paragordius* (Szmygiel et al. 2014; Swanteson-Franz et al. 2018). Although the larval morphology conformed to the typical *Gordius* larval type, the three pairs of left, right, and dorsal spines on the distal end of the proboscis differed from the only other SEM imaged proboscis of an undescribed species of Gordius cf. robustus collected from streams in New Mexico (clade 3 in Hanelt et al. 2015). Szmygiel et al. (2014) reported that the right and left lateral sides of the proboscis of the New Mexico G. cf. robustus contained four pairs of spines; whereas the dorsal side contained three pairs of spines, all arranged in tandem. Finally, the egg morphology of *G. terrestris* sp. nov. was unlike egg descriptions for any other hairworm species (Schmidt-Rhaesa 1997a; Adrianov et al. 1998; Marchiori et al. 2009; Szmygiel et al. 2014; Bolek et al. 2015). Eggs of *G. terrestris* sp. nov. contained an outer shell separated by distinct space from a thick inner membrane. Our evaluation of eggs of three aquatic species of Gordiids (*G. difficilis*, *N. occidentalis*, and *P. obamai*) indicate that their eggs are elliptical in shape, with a distinct shell and a thin inner membrane surrounding the developing larva (Fig. 7).

## Discussion

*Gordius terrestris* sp. nov. represents the first hairworm species consistently collected from a terrestrial habitat. Hundreds of adult free-living worms appeared after bouts of heavy rain on streets, sidewalks, and lawns during the winter season, where male and female worms were observed mating and some females were observed depositing egg strings (unpublished data). It is currently unclear what definitive host is used in the life cycle of *G. terrestris* sp. nov. However, over a two-year sampling period, no arthropod hosts were observed in the areas when adult worms appeared. More intriguing, free-living adult worms would disappear from these locations within days after the rains subsided.

Currently, there is only one other report of a European gordiid depositing egg strings in a terrestrial habitat. Schmidt-Rhaesa (2013) reported female *Gordius aquaticus* laying eggs under moist rotting leaves directly adjacent to water; whereas males of this species were observed in shallow forest streams and ponds. In contrast to the *G. aquatics* observations, all collections of adult *G. terrestris* sp. nov. in this study and our previous collections of this species from Oklahoma, Texas, and Louisiana in Hanelt et al. (2015) and Harkins et al. (2016) were from terrestrial habitats. Finally, field surveys by Harkins et al. (2016) for hairworm cysts in aquatic paratenic hosts from 46 streams in Payne Co. Oklahoma, including the City of Stillwater, indicate that *Gordius* type cysts accounted for 1.7 % (31/1,749) of the total cysts collected, compared to 98.3% of cysts being represented by aquatic hairworm species in the genera *Paragordius*, *Chordodes*, and/or *Neochordodes* where they commonly mate. This is particularly significant since adults of *G. terrestris* sp. nov. is the most commonly encountered gordiid on lawns and sidewalks by the public in Oklahoma and Texas (MGB unpublished data) suggesting that *G. terrestris* sp. nov. is commonly encountered in terrestrial habitats and nonadult stages are rarely found in aquatic habitats.

One significant observation is the unique egg morphology of *G. terrestris* sp. nov. with a thick inner membrane surrounding the developing larval stage. Although few detailed hairworm egg descriptions or egg photographs exist in the literature, our evaluation of eggs for three aquatic gordiid species, clearly indicate that in aquatic Gordiids the developing larval stage is surrounded by a thin inner membrane (Schmidt-Rhaesa 1997b; Bolek and Coggins 2002; Bolek et al. 2010, 2013a, 2015; Schmidt-Rhaesa 2013). Additionally, our unpublished observations on the egg morphology of three undescribed *Gordius* species collected from aquatic habitats in Nebraska, New Mexico, and California (clades 2, 3, and 4 in Hanelt et al. 2015) also indicate that the eggs of these aquatic species lack the unique egg morphology of *G. terrestris* sp. nov. Considering the terrestrial habitat free-living adult *G. terrestris* sp. nov. occur in, we hypothesize that this unique egg morphology may be an adaptation for terrestrial habitats. In the future, we plan to publish our detailed observations on mating and oviposition by this species in terrestrial environments and the occurrence of cysts of *G. terrestris* sp. nov. in terrestrial paratenic hosts.

## Supplementary Material

XML Treatment for Gordius terrestris
